# On the stability of nucleoside diphosphate glucose metabolites: implications for studies of plant carbohydrate metabolism

**DOI:** 10.1093/jxb/erx190

**Published:** 2017-06-13

**Authors:** Benjamin L Hill, Carlos M Figueroa, Matías D Asencion Diez, John E Lunn, Alberto A Iglesias, Miguel A Ballicora

**Affiliations:** 1 Department of Chemistry and Biochemistry, Loyola University Chicago, 1068 West Sheridan Road, Chicago, IL, USA; 2 Instituto de Agrobiotecnología del Litoral, UNL, CONICET, FBCB, Colectora Ruta Nacional 168 km 0, Santa Fe, Argentina; 3 Max Planck Institute of Molecular Plant Physiology, Am Muehlenberg, Potsdam-Golm, Germany

**Keywords:** ADP-glucose, NMR, plastid, starch, sucrose, UDP-glucose

## Abstract

Nucleoside diphosphate sugars (NDP-sugars) are the substrates for biosynthesis of oligo- and polysaccharides, such as starch and cellulose, and are also required for biosynthesis of nucleotides, ascorbic acid, several cofactors, glycoproteins and many secondary metabolites. A controversial study that questions the generally accepted pathway of ADP-glucose and starch synthesis in plants is based, in part, on claims that NDP-sugars are unstable at alkaline pH in the presence of Mg^2+^ and that this instability can lead to unreliable results from *in vitro* assays of enzyme activities. If substantiated, this claim would have far-reaching implications for many published studies that report on the activities of NDP-sugar metabolizing enzymes. To resolve this controversy, we investigated the stability of UDP- and ADP-glucose using biophysical, namely nuclear magnetic resonance (NMR), and highly specific enzymatic methods. Results obtained with both techniques indicate that NDP-sugars are not as unstable as previously suggested. Moreover, their calculated *in vitro* half-lives are significantly higher than estimates of their *in planta* turnover times. This indicates that the physico-chemical stability of NDP-sugars has little impact on their concentrations *in vivo* and that NDP-sugar levels are determined primarily by the relative rates of enzymatic synthesis and consumption. Our results refute one of the main arguments for the controversial pathway of starch synthesis from imported ADP-glucose produced by sucrose synthase in the cytosol.

## Teaser

Biophysical and enzymatic analyses show that UDP- and ADP-glucose are more stable than claimed in a controversial study that questions the generally accepted pathway of starch synthesis in plants.

## Introduction

Nucleoside diphosphate sugars (NDP-sugars) were discovered by Luis F Leloir and colleagues around the middle of the twentieth century (Leloir, 1951). This seminal discovery and the complementary work on enzyme characterization and elucidation of the many critical roles played by NDP-sugars in cells, led to Leloir being awarded the Nobel Prize in Chemistry in 1970 (Leloir, 1971; Leloir, 1983). Early studies determined that UDP-Glc is a key intermediate involved in the metabolism of galactose (Leloir, 1951) and is the glucosyl donor for trehalose synthesis in yeast (Leloir and Cabib, 1953), sucrose synthesis in plants (Cardini *et al.*, 1955) and the elongation of α-1,4-polyglucan chains during glycogen synthesis in mammals (Leloir *et al.*, 1959). UDP-Glc is also the substrate for cellulose synthesis and an intermediate in the production of other cell wall polymers (Carpita, 2011). Other NDP-sugars and NDP-sugar metabolizing enzymes were subsequently discovered, with the first report of an ADP-Glc synthesizing enzyme coming from soybean (Espada, 1962). Later studies showed that ADP-Glc, not UDP-Glc, is the glucosyl donor for glycogen synthesis in bacteria (Greenberg and Preiss, 1964) and starch synthesis in plants (Murata *et al.*, 1963; Recondo *et al.*, 1963; Ghosh and Preiss, 1966).

Starch is the most important carbon reserve in plants and the major source of calories in our staple crops. Transitory starch reserves are accumulated and degraded on a daily basis in the leaves of many plants to provide carbon and energy during the hours of darkness. Starch is also stored in many seeds, fruits, and tubers, as well as in the stems of woody perennials, providing longer term reserves for germination and regrowth after winter. In leaves, starch is synthesized in the chloroplasts from fructose 6-phosphate that is withdrawn from the Calvin-Benson cycle and converted to ADP-Glc via plastidial phosphoglucose isomerase (pPGI), plastidial phosphoglucomutase (pPGM), and ADP-Glc pyrophosphorylase (ADP-Glc PPase), which is restricted to the chloroplasts in leaves (Ballicora *et al.*, 2004). In heterotrophic tissues, starch is made in specialized amyloplasts, in most cases via plastidial ADP-Glc PPase using imported hexose-phosphates. An exception is cereal endosperm, where additional cytosolic isoforms of ADP-Glc PPase are responsible for the majority of ADP-Glc production, with ADP-Glc being imported into the amyloplasts via a BRITTLE1-type nucleotide transporter for starch synthesis (Comparot-Moss and Denyer, 2009).

The classical pathway is supported by the demonstrated autonomy of illuminated chloroplasts to synthesize starch from CO_2_ (Gibbs and Cynkin, 1958; Heldt *et al.*, 1977) and by the starch deficient phenotypes of *pgi*, *pgm* and *adg* mutants lacking pPGI, pPGM and ADP-Glc PPase, respectively (Caspar *et al.*, 1985; Lin *et al.*, 1988; Yu *et al.*, 2000). Despite the considerable biochemical and genetic evidence supporting the ADP-Glc PPase-mediated pathway, its contribution to starch synthesis has been disputed by Pozueta-Romero and colleagues. They have proposed an alternative pathway in which ADP-Glc is produced by sucrose synthase (SUS) in the cytosol and then imported into plastids via a hypothetical ADP-Glc transporter (Baroja-Fernández *et al.*, 2001). The initial version of the proposed SUS-mediated pathway was criticized because it did not explain the starch-deficient phenotypes of mutants lacking ADP-Glc PPase or pPGM (Neuhaus *et al.*, 2005). To counter this criticism, Pozueta-Romero and colleagues postulated that starch synthesis is always accompanied by futile cycling of starch. They propose that starch is synthesized initially from imported ADP-Glc, but is constantly turned over via hydrolytic pathways that yield Glc and Glc-1P, and that plastidial hexokinase, pPGM and ADP-Glc PPase are needed to salvage these metabolites to resynthesize starch (Baroja-Fernández *et al.*, 2001; Baroja-Fernández *et al.*, 2004; Baroja-Fernández *et al.*, 2005; Bahaji *et al.*, 2011; Bahaji *et al.*, 2014*a*,*b*; Baslam *et al.*, 2017). More recently, these authors proposed that the starch deficient phenotype of *pgi* mutants is the result of reduced photosynthetic capacity, most likely as a consequence of reduced levels of plastidial cytokinins (Bahaji *et al.*, 2015). In agreement with this hypothesis, Sánchez-López *et al.* (2016) showed that *pgi* mutants accumulate high levels of starch when exposed to volatile emissions from the fungus *Alternaria alternata*, which promote photosynthesis, growth, and accumulation of plastidic cytokinins.

The proposed SUS-mediated pathway and associated futile cycling of starch remains highly controversial (Neuhaus *et al.*, 2005; Stitt and Zeeman, 2012; Streb and Zeeman, 2012) and several reverse genetic studies have been undertaken to test its validity. *Arabidopsis thaliana* mutants lacking key enzymes of starch degradation were crossed with a *pgm* mutant lacking pPGM activity (Streb *et al.*, 2009). If the alternative SUS-mediated pathway and futile cycling of starch were operational, the double mutants would be expected to accumulate high levels of starch, but in fact all of them had a starch-deficient phenotype similar to the parental *pgm* plants (Streb *et al.*, 2009). The epistatic effect of the *pgm* mutation is entirely consistent with the classical pathway. Arabidopsis has six *SUS* genes (Bieniawska *et al.*, 2007), of which *AtSUS1–AtSUS4* are expressed in leaf mesophyll cells where transitory starch is accumulated, while expression of *AtSUS5* and A*tSUS6* is restricted to the developing vascular tissue (Barratt *et al.*, 2009). A quadruple *sus1sus2sus3sus4* (*sus1234*) mutant that lacks SUS expression in mesophyll cells was found to accumulate wild-type levels of starch (Barratt *et al.*, 2009). Although this observation was confirmed by Baroja-Fernández *et al.* (2012*a*), they claimed that Bieniawska *et al.* (2007) and Barratt *et al.* (2009) had underestimated SUS activity in the *sus* mutants and that the *sus1234* mutant retained sufficient SUS activity to account for the observed wild-type rates of starch accumulation. This criticism was in part based on a claim that UDP-Glc would be unstable in the presence of Mg^2+^ under the mildly alkaline conditions of pH 9.4 of the assay used by Bieniawska *et al.* (2007) and Barratt *et al.* (2009), leading to loss of UDP-Glc during the assay and underestimation of SUS activity. The authors of the latter study responded that even if the residual SUS activity had been underestimated, this did not affect the main conclusion of Barratt *et al.* (2009) that knocking out all four of the *SUS* genes expressed in mesophyll cells had no discernible effect on starch accumulation (Smith *et al.*, 2012). However, the question of whether the residual SUS activity in the *sus1234* mutant was underestimated due to instability of UDP-Glc in the *in vitro* assay has not yet been addressed.

It has been known for many years that UDP-Glc spontaneously undergoes cleavage in mildly alkaline conditions to produce UMP and the cyclic monophosphoric ester Glc 1,2-phosphate (Paladini and Leloir, 1952). This process requires Mg^2+^ (Zervosen *et al.*, 1998) and has also been described for ADP-Glc (Murata *et al.*, 1964). These observations appear to support the claims of Baroja-Fernández *et al.* (2012*a*) that SUS activities were underestimated by Bieniawska *et al.* (2007) and Barratt *et al.* (2009). They also imply that the activities of UDP-Glc or ADP-Glc metabolizing enzymes could have been underestimated in many other previous studies, if inappropriate conditions had been used for *in vitro* assays. If substantiated, the proposed instability of NDP-sugars could also affect our understanding of their metabolism *in vivo*. Illumination of leaves leads to alkalinization of the chloroplast stroma and release of Mg^2+^ from the thylakoids, leading to a steady state pH of about 8.3 and a Mg^2+^ concentration of about 6 mM (Werdan *et al.*, 1975; Portis and Heldt, 1976; Krause, 1977; Portis, 1981). It has been claimed that ADP-Glc would be highly unstable under such conditions, implying that the chemical environment of the stroma of illuminated chloroplasts is unfavourable for ADP-Glc synthesis (Baroja-Fernández *et al.*, 2001).

The aim of the current study was to determine the pH sensitivity of UDP-Glc and ADP-Glc in the presence or absence of Mg^2+^. This will not only address the specific question of whether SUS activity could have been underestimated in the studies of Bieniawska *et al.* (2007) and Barratt *et al.* (2009), but also whether alkaline sensitivity of NDP-sugars could have compromised other studies of NDP-sugar metabolizing enzymes. Results presented in this work are critical for the proper study of all glycosyltransferases, not only in plants but also in other organisms. They are also relevant for biotechnological processes (De Bruyn *et al.*, 2015), such as glycorandomization and *in vitro* production of glycoproteins, where the physico-chemical stability of NDP-sugar reactants could be a significant constraint.

We used biophysical, namely nuclear magnetic resonance (NMR), and enzymatic methods to investigate the stability of UDP-Glc and ADP-Glc over a range of pH and Mg^2+^ concentrations spanning the conditions used in the disputed *in vitro* assays of SUS activity. We determined the half-life of these UDP-sugars in conditions that simulate the *in vivo* environment of the stroma in illuminated chloroplasts and in other subcellular compartments.

## Materials and methods

### Chemicals and enzymes

UDP-Glc, ADP-Glc, UMP, AMP, sodium pyrophosphate, NADP^+^, Glc 1,6-bisphosphate, phosphoglucomutase from rabbit muscle, Glc-6-phosphate dehydrogenase from Baker’s yeast, BSA and MgCl_2_ were obtained from Sigma-Aldrich (St. Louis, MO, USA). Deuterium oxide was from Cambridge Isotope Laboratories Inc. (Andover, MA, USA). All other chemicals were of the highest quality available. Recombinant ADP-Glc and UDP-Glc pyrophosphorylases from *Escherichia coli* were expressed in *E. coli* and purified to near homogeneity as previously described (Ballicora *et al.*, 2002; Ebrecht *et al.*, 2015).

### Sample preparation and NMR analysis

Samples 1 ml in volume containing 5 mM UDP-Glc, 10 mM MgCl_2_, 10% deuterium oxide, and 30 mM of either HEPES-NaOH at pH 7.0, BisTris propane-NaOH at pH 8.0, or CHES-NaOH at pH 8.5 and 9.0, were incubated in a 37°C water bath for 0, 12, 20, 45, or 90 min. All pH measurements were made at 37°C. Additionally, a 1 ml sample containing 5 mM ADP-Glc, 10 mM MgCl_2_, 10% deuterium oxide, and 30 mM CHES-NaOH at pH 9.0 was incubated in a 37°C water bath for 90 min. Once removed from the water bath, 250 μl of a solution containing 1 M HEPES-NaOH at pH 7.0 and 150 mM EDTA were added to the 1 ml samples. The neutral pH and chelation of Mg^2+^ by excess EDTA prevented further degradation of the NDP-Glc (Zervosen *et al.*, 1998). Samples were immediately frozen with liquid nitrogen and kept at -80°C until use. After thawing, [^31^P]NMR was performed on these samples to quantify the extent of NDP-sugar degradation that occurred during the incubation period. Experiments determining the relative NDP-Glc, nucleoside monophosphate (NMP), and cyclic Glc 1,2-phosphate concentrations were performed using a Varian Inova 500 MHz NMR spectrometer. A dilute phosphoric acid sample served as the external standard, denoting 0 ppm. At least 6000 scans were performed on all samples using a 33° tip angle and a 3.2 s acquisition time, while at least 12 000 scans were performed on samples with more than 80% apparent NDP-Glc degradation. The relative integration values of the observed species were used to calculate the percentage of NDP-Glc degradation that occurred during the given time period. As NMPs are known to be stable in alkaline solutions containing Mg^2+^ (Nanninga, 1957), but the stability of cyclic Glc 1,2-phosphate is not known, calculations for the percentage of degradation were based on the integration values of the NMP and NDP-Glc using the following equation: (2×*A*_NMP_)/[(2×*A*_NMP_)+*A*_NDP-Glc_]×100, where *A*_NMP_ is the integrated area for the NMP and *A*_NDP-Glc_ is the area of NDP-Glc. Half-lives for UDP-Glc in each of the pH conditions were calculated using the formula: *P*=(1−2^−t/z^)×100, where *P* is the percentage of UDP-Glc degradation, *t* is time in minutes, and *z* is the calculated half-life.

The chemical shift peaks of NMP and NDP-Glc were determined from standard solutions. Each 5 mM solution was prepared with 30 mM HEPES-NaOH at pH 7.0, 10 mM MgCl_2_, and 10% deuterium oxide, to which 250 μl of a solution containing 1 M HEPES-NaOH at pH 7.0 and 150 mM EDTA were added. Peaks were found to be near 3 ppm for the NMP and near -11 and -13 ppm for the two phosphates of the NDP-Glc. Consistent with previously published results (Withers *et al.*, 1981), we concluded that the peak observed near 11 ppm in our experimental samples belongs to cyclic Glc 1,2-phosphate.

To determine the spin-lattice relaxation times (*T*_*1*_), experiments of *T*_*1*_ inversion recovery were performed as previously described (Günther, 2013). A standard containing UDP-Glc, UMP, and cyclic Glc 1,2-phosphate was derived from a sample originally containing 10 mM UDP-Glc, 20 mM MgCl_2_, 10% deuterium oxide, and 30 mM CHES-NaOH at pH 9.0 which was incubated at 37°C for 2 h. After incubation, 250 μl of 1 M HEPES-NaOH at pH 7.0, with 150 mM EDTA, were added prior to NMR analysis to stop further NDP-sugar degradation. The longest *T*_*1*_ time of any analyte (UDP-Glc, UMP, or cyclic Glc 1,2-phosphate) was 3.3 s. As described elsewhere (Ernst and Anderson, 1966), the use of a 33° tip angle reduces this by a factor of 5.56, yielding an effective *T*_*1*_ time of 0.59 s. Therefore, when a 33° tip angle is used, an acquisition time of 3.2 s is greater than 5×*T*_*1*_ of any analyte being quantified. In addition, the *T*_*1*_ relaxation times for UDP-Glc that we observed matched well with previous research (Wehrli *et al.*, 1992).

### Enzymatic determination of NDP-sugars degradation

Four 1 ml solutions, each containing 0, 1.25, 2.5, or 5 mM NDP-Glc, 10 mM MgCl_2_ and 30 mM CHES-NaOH at pH 9.0, were incubated in a water bath at 37°C. The reaction was started by the addition of the NDP-sugar. After 90 min, 250 µl of 1 M HEPES-NaOH at pH 7.0 was added to achieve neutralization and to stop any further degradation of the NDP-sugar. Neutralization was confirmed by using pH indicator paper. Separately, four replicate ‘time zero’ samples were prepared in the same way, except that the 250 µl of 1 M HEPES-NaOH at pH 7.0 was added before the NDP-Glc and the reaction mixture was not incubated at 37°C. Following neutralization, the amount of intact NDP-Glc remaining in each of these solutions was enzymatically determined as previously described (Munch-Petersen, 1955; Sowokinos, 1976), with the following modifications: a 10-µl aliquot of neutralized sample was added to 160 µl of a solution containing 200 mM HEPES-NaOH at pH 7.3, 5 mM MgCl_2_, 1.5 mM sodium pyrophosphate, 10 mM sodium fluoride, 1.25 mM NADP^+^, 2 mM dithiothreitol, 0.01 mM Glc 1,6-bisphosphate, 3 U/ml phosphoglucomutase, 3 U/ml Glc-6-phosphate dehydrogenase, 0.2 mg/ml BSA, and 1 U of either UDP-Glc pyrophosphorylase or ADP-Glc PPase. The conversion of NADP^+^ to NADPH was monitored using a BioTek EL808 microplate reader (Winooski, VT, USA) measuring the absorbance at 340 nm every 15 s. The absorbance was measured for at least 4 min after there was no further change to ensure that the reaction had reached completion, resulting in measurements spanning approximately 8 min in total. The absorbance value of the blank samples without NDP-Glc was subtracted from all readings.

## Results and discussion

**Fig. 1.**
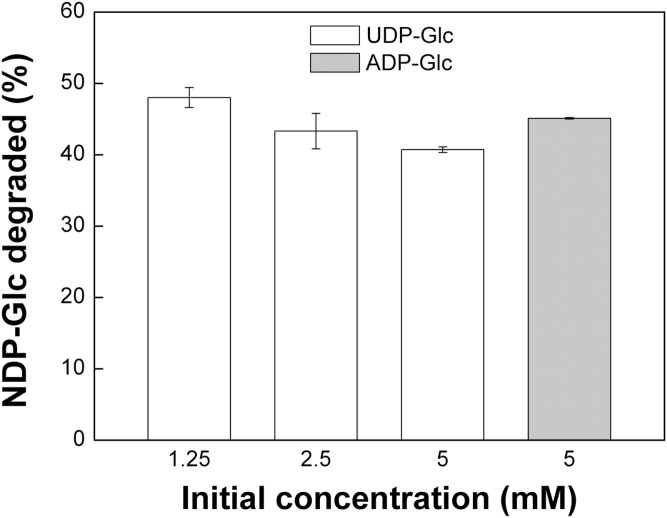
Extent of NDP-Glc degradation determined using the enzymatic method. The results match well with those obtained from the NMR analysis and also show that the rate of degradation is marginally dependent on the initial concentration of the NDP-sugar.

The stability of UDP-Glc and ADP-Glc was initially assessed by incubation of known amounts at pH 9.0 and 37°C, in the presence of 10 mM MgCl_2_, and measuring the amount remaining after 90 min by enzymatic analysis with UDP-Glc pyrophosphorylase and ADP-Glc PPase, respectively. These conditions were chosen as a physiologically extreme scenario, combining high pH and high Mg^2+^ concentration with high temperature. Under these extreme conditions, 40–48% of the UDP-Glc was degraded within 90 min (Fig. 1), with only a weak dependence on the initial concentration (Fig. 1). There was a similar loss of 45% of ADP-Glc under these conditions (Fig. 1).

**Fig. 2.**
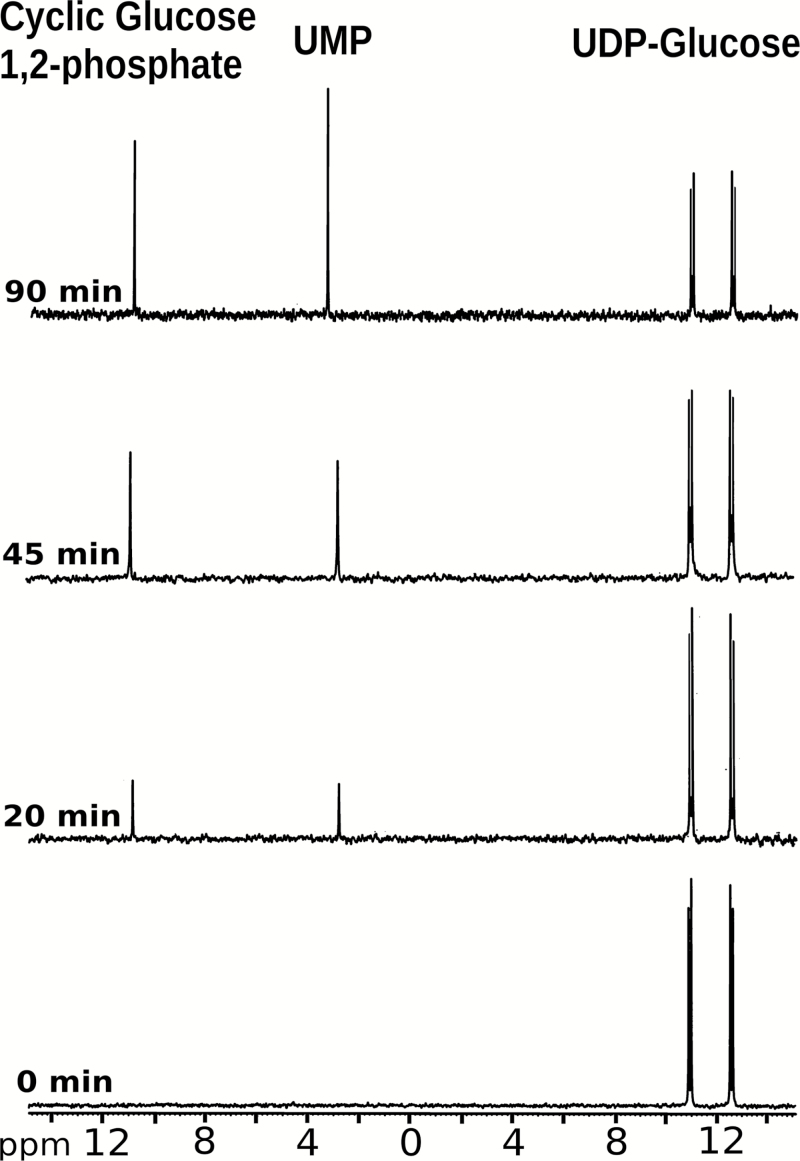
NMR spectra of UDP-Glc samples incubated for 0, 20, 45, and 90 min at pH 9.0 with 10 mM Mg^2+^. The time-dependent cleavage of UDP-Glc gives rise to UMP and cyclic Glc 1,2-phosphate.

**Fig. 3.**
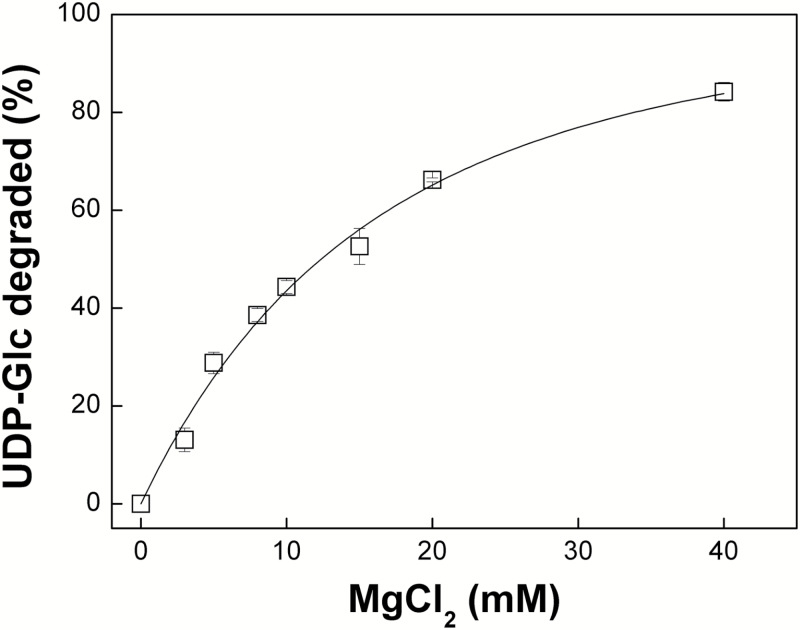
Degradation of UDP-Glc at pH 9.0 and varying concentrations of Mg^2+^. Degradation of the NDP-sugar depends on the concentration of the divalent cation.

**Fig. 4.**
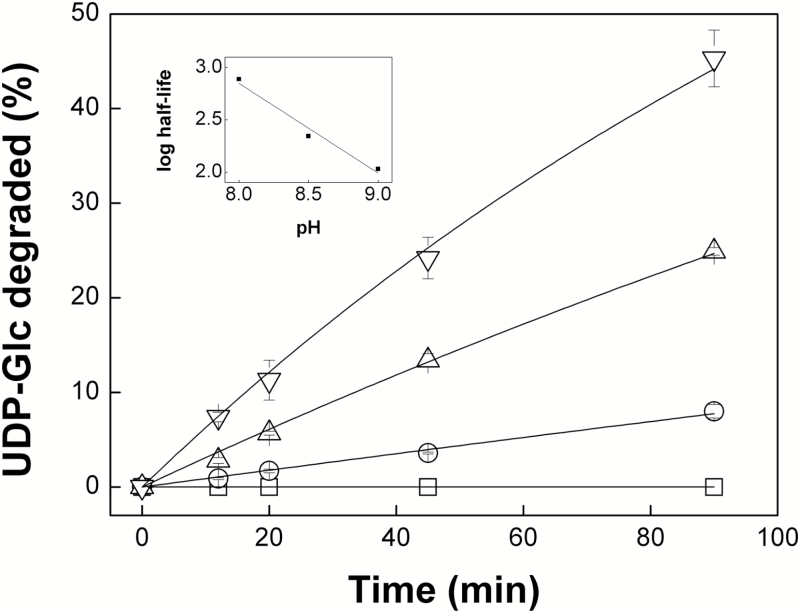
Degradation of UDP-Glc is higher at alkaline pH values. The main plot shows data derived from the NMR analysis of UDP-Glc samples that were incubated for 0, 12, 20, 45, and 90 min with 10 mM Mg^2+^ at pH 7.0 (square), 8.0 (circle), 8.5 (triangle) and 9.0 (inverted triangle). These curves were used to calculate the half-lives of UDP-Glc at different pH values. The inset shows the linear correlation between the log_10_ of the UDP-Glc half-lives and the pH.

We used [^31^P]NMR to investigate the kinetics and dependence of the degradation on pH and Mg^2+^ concentration in more detail. When incubated at pH 9.0 in the presence of 10 mM MgCl_2_, the amplitude of the UDP-Glc chemical shift peaks decreased over time and was accompanied by the appearance of additional peaks corresponding to the chemical shifts of UMP and cyclic Glc 1,2-phosphate (Fig. 2), the characteristic degradation products from alkaline cleavage of UDP-Glc (Paladini and Leloir, 1952). The cleavage process required the presence of Mg^2+^, with the amount of UDP-Glc degraded showing a first order exponential response to increasing Mg^2+^ concentration, resulting in 84% degradation at 40 mM Mg^2+^ (Fig. 3). No detectable loss of UDP-Glc was observed at pH 7.0 but degradation was detectable at pH 8.0 and increased at higher pH values up to pH 9.0 (Fig. 4). Calculated half-lives for UDP-Glc were 773 min at pH 8.0, 220 min at pH 8.5, and 107 min at pH 9.0. There was a linear correlation between the log_10_ of the half-lives for UDP-Glc and the pH (Pearson’s *r*=-0.988), suggesting that the reaction depends on the concentration of hydroxyl ions (Fig. 4, inset). Degradation of ADP-Glc reached 47% after 90 min incubation at pH 9.0 with 10 mM Mg^2+^, a similar value to that observed for UDP-Glc (Fig. 4).

These results confirm the previous reports from Paladini and Leloir (1952) and Murata *et al.* (1964) that UDP-Glc and ADP-Glc decompose under alkaline conditions. However, their susceptibility to degradation is strongly dependent on pH and Mg^2+^ concentration (Figs 3 and 4), and even under the most extreme conditions tested, namely pH 9.0, 10 mM MgCl_2_ and 37°C, over 50% of the initial UDP-Glc and ADP-Glc survived incubation for 90 min (Figs 1 and 4). In plant cells, UDP-Glc is located predominantly, or even exclusively, in the cytosol (Szecowka *et al.*, 2013; Arrivault *et al.*, 2014), which typically has a pH of 7.3–7.7 (Ishizawa, 2014) and a concentration of free Mg^2+^ of 0.2–0.4 mM (Yazaki *et al.*, 1988; Gout *et al.*, 2014). From our results, we can conclude that UDP-Glc is stable under such conditions. Its concentration *in vivo* will therefore be determined by the relative rates of UDP-Glc synthesis and consumption by UDP-Glc metabolizing enzymes, with its chemical instability at alkaline pH having little or no significant impact on *in vivo* levels.

During photosynthesis, ion movements across the thylakoid membranes result in alkalinization of the stroma to around pH 8.3 and a rise in Mg^2+^ concentration to around 6 mM (Werdan *et al.*, 1975; Portis and Heldt, 1976; Krause, 1977; Portis, 1981). Although these conditions appear to be more favourable to chemical cleavage of NDP-Glc metabolites, in reality the impact of this instability is likely to be very limited. The calculated half-lives for UDP-Glc at alkaline pH values ranged from 107–773 min, which is over two orders of magnitude higher than the estimated turnover time for UDP-Glc of 36 s *in planta* (Arrivault *et al.*, 2009). ADP-Glc displayed similar chemical stability to UDP-Glc (Figs 1 and 4); therefore, we can reasonably expect half-life values for ADP-Glc to be in the same range as UDP-Glc. Such values would be over four orders of magnitude greater than the estimated turnover time for ADP-Glc of 0.5 s *in planta* (Arrivault *et al.*, 2009). Thus, our data do not support the idea that the chemical instability of ADP-Glc favors its accumulation in the cytosol rather than the chloroplast stroma, which was a theoretical argument put forward to support the alternative pathway of starch biosynthesis (Baroja-Fernández *et al.*, 2001; Bahaji *et al.*, 2011). We conclude that the chemical instability of UDP-Glc and ADP-Glc under mildly alkaline conditions has no significant impact on their concentrations *in vivo*.

Our results also demonstrate that even under the most extreme conditions tested, namely pH 9.0, 10 mM Mg^2+^ and 37°C, losses of UDP-Glc and ADP-Glc were less than 50% after 90 min incubation. The disputed SUS activity data reported by Bieniawska *et al.* (2007) and Barratt *et al.* (2009) came from *in vitro* assays with reaction mixtures containing 2.2 mM MgCl_2_, which were incubated at 20°C for only 20 min. Based on the data (Figs 3 and 4), we can estimate that the difference in Mg^2+^ concentration alone would reduce the loss of UDP-Glc by a factor of three compared with the most extreme conditions tested, and that the shorter incubation time of 20 min versus 90 min would reduce losses by a further factor of 4.5. Thus, the maximal loss of UDP-Glc to be expected under the assay conditions used by Bieniawska *et al.* (2007) and Barratt *et al.* (2009) would lead to underestimation of SUS activity by less than 10%. In reality, losses of UDP-Glc would be even lower because of the lower incubation temperature of 20°C versus 37°C. From these calculations, the instability of UDP-Glc would have had little impact on the SUS activities measured by Bieniawska *et al.* (2007) and Barratt *et al.* (2009), with the reported values underestimating the true activity by around 10% at most and very likely even less. The discrepancies between the various reports of SUS activities in *sus* mutants (Bieniawska *et al.* 2007; Barratt *et al.*, 2009; Baroja-Fernández *et al.*, 2012*a*) remain to be resolved. However, the criticism by Baroja-Fernández *et al.* (2012*a*) of the SUS activity data in the other two studies, based on the assumed instability of UDP-Glc under the *in vitro* assay conditions, is not supported by our results. It is also worth reiterating that even if residual SUS activity in the quadruple *sus1234* mutant was underestimated by Barratt *et al.* (2009), none of this activity would be in the mesophyll cells where starch synthesis occurs (Barratt *et al.* 2009; Smith *et al.*, 2012). Wild-type levels of starch in the quadruple loss-of-function mutant therefore indicate that SUS makes no significant contribution to starch synthesis in Arabidopsis leaves.

In conclusion, the chemical instability of UDP-Glc and ADP-Glc at alkaline pH in the presence of Mg^2+^ is unlikely to have any significant impact on *in vivo* concentrations of these metabolites in plants. Arguments in favor of the alternative pathway of starch synthesis based on NDP-sugar instability therefore have little merit.

## Author contributions

AAI and MAB conceived the project; BLH, CMF and MDAD performed experiments; all authors analyzed data and wrote the manuscript.

## Abbreviations:

ADP-Glc PPase: ADP-glucose pyrophosphorylase
NDP: nucleoside diphosphate
NMP: nucleoside monophosphate
pPGI: plastidial phosphoglucose isomerase
pPGM: plastidial phosphoglucomutase.
